# American Medical Association

**Published:** 1848-05

**Authors:** 


### AMERICAN MEDICAL ASSOCIATION.

Professor Yandell has been appointed by the Medical Faculty of the University of Louisville a delegate to the American Medical Association, which meets in Baltimore on Tuesday, the 2d of May. The proceedings of the Association will no doubt possess unusual interest, and we have made arrangements for communicating them to our readers at the earliest day. We hope to be able to give full details in our next number.

